# Supplementary material for: Comparative assessment of the feasibility and validity of daily activity space in urban and non-urban settings
Source: PLoS One. 2024 Jan 30;19(1):e0297492. doi: 10.1371/journal.pone.0297492 (PMC10826934; doi:10.1371/journal.pone.0297492)
Supplement: S1 Table — GPS quality metrics and incidence of outliers by participants’ residential and activity space locations. (DOCX) [file pone.0297492.s001.docx]

| **S1 Table. Summary statistics for GPS quality metrics and incidence of outliers by participants’ residential and activity space locations: Android devices only** | | | | | |
| --- | --- | --- | --- | --- | --- |
|  | Urban residence^†^ | | Non-Urban residence^†^ | | All |
|  | Urban location* | Non-Urban location* | Urban location* | Non-Urban location* | Android |
| Time gap |  |  |  |  |  |
| Mean | 4.917 | 2.051 | 1.279 | 3.085 | 4.204 |
| SD | 233.315 | 25.556 | 59.586 | 187.895 | 214.154 |
| Median | 1 | 1 | 1 | 1 | 1 |
| % outlier (>1 hour) | 0.012% | 0.001% | 0.003% | 0.007% | 0.010% |
| Distance gap |  |  |  |  |  |
| Mean | 5.918 | 13.330 | 12.666 | 10.196 | 7.576 |
| SD | 71.802 | 122.322 | 61.599 | 37.752 | 66.325 |
| Median | 0 | 2.751 | 6.610 | 1.010 | 0 |
| % outlier (>1 km) | 0.025% | 0.040% | 0.018% | 0.008% | 0.021% |
| Accuracy |  |  |  |  |  |
| Mean | 18.447 | 13.253 | 14.060 | 18.640 | 18.228 |
| SD | 57.009 | 49.519 | 82.833 | 63.840 | 59.454 |
| Median | 14.26 | 5.42 | 5.14 | 14.02 | 13.23 |
| % outlier (>1 km) | 0.027% | 0.102% | 0.140% | 0.049% | 0.039% |
|  |  |  |  |  |  |
| N (GPS points) | 4,827,073 | 271,725 | 144,632 | 2,177,558 | 7,420,988 |
| % GPS points by location | 94.671% | 5.329% | 6.228% | 93.772% | - |
| ^†^ Location of participant’s residence. | | | | | |
| * Location where GPS reading was taken. | | | | | |
