# Supplementary material for: Comparative assessment of the feasibility and validity of daily activity space in urban and non-urban settings
Source: PLoS One. 2024 Jan 30;19(1):e0297492. doi: 10.1371/journal.pone.0297492 (PMC10826934; doi:10.1371/journal.pone.0297492)
Supplement: S2 Table — GPS quality metrics and incidence of outliers by participants’ residential and activity space locations. (DOCX) [file pone.0297492.s002.docx]

| **S2 Table. Summary statistics for GPS quality metrics and incidence of outliers by participants’ residential and activity space locations: iOS devices only** | | | | | |
| --- | --- | --- | --- | --- | --- |
|  | Urban residence^†^ | | Non-Urban residence^†^ | | All |
|  | Urban location* | Non-Urban location* | Urban location* | Non-Urban location* | iOS |
| Time gap |  |  |  |  |  |
| Mean | 1.867 | 1.680 | 1.304 | 1.867 | 1.851 |
| SD | 144.148 | 79.361 | 38.245 | 143.650 | 141.561 |
| Median | 1 | 1 | 1 | 1 | 1 |
| % outlier (>1 hour) | 0.004% | 0.004% | 0.001% | 0.004% | 0.004% |
| Distance gap |  |  |  |  |  |
| Mean | 1.935 | 7.978 | 9.050 | 1.817 | 2.182 |
| SD | 27.965 | 65.665 | 53.109 | 32.093 | 30.945 |
| Median | 0 | 0 | 0.333 | 0 | 0 |
| % outlier (>1 km) | 0.006% | 0.023% | 0.028% | 0.008% | 0.007% |
| Accuracy |  |  |  |  |  |
| Mean | 9.272 | 9.401 | 11.924 | 9.352 | 9.350 |
| SD | 249.399 | 114.893 | 34.063 | 33.584 | 213.156 |
| Median | 4.77 | 4.75 | 5.07 | 4.76 | 4.77 |
| % outlier (>1 km) | 0.015% | 0.034% | 0.010% | 0.007% | 0.014% |
|  |  |  |  |  |  |
| N (GPS points) | 22,703,510 | 639,338 | 669,620 | 7,456,060 | 31,468,528 |
| % GPS points by location | 97.261% | 2.739% | 8.241% | 91.759% | - |
| ^†^ Location of participant’s residence. | | | | | |
| * Location where GPS reading was taken. | | | | | |
