# Supplementary material for: Comparative assessment of the feasibility and validity of daily activity space in urban and non-urban settings
Source: PLoS One. 2024 Jan 30;19(1):e0297492. doi: 10.1371/journal.pone.0297492 (PMC10826934; doi:10.1371/journal.pone.0297492)
Supplement: Attachment — Submitted filename: Response to Reviewers.docx [file pone.0297492.s003.docx]

[Reuben-Cooke Building](https://maps.duke.edu/?focus=207)	[417 Chapel Drive](https://maps.duke.edu/?focus=207)

Campus Box 90086

Duke University

Durham, NC 27708	psychneuro@duk[e.edu](mailto:psychneuro@duke.edu)

21 December 2023

Abel C. H. Chen

Academic Editor

*PLOS ONE*

Dear Dr. Chen:

Thank you for managing the review of our manuscript [PONE-D-23-31641] and for the invitation to submit a revised version. We sincerely appreciate the comments and suggestions, all of which we have addressed in the revised manuscript. We believe the revised manuscript is stronger and clearer as a result.

Below we describe the revisions we made in response to the suggestions provided by you and the reviewers. The original comments are listed first; our responses follow in blue text. Within the manuscript, you will find tracked changes showing the edits and revisions, including new text on previous work in urban spaces, which we believe highlights the importance of our work.

Please update our financial disclosure statement to read as follows:

This work was funded by the National Institute on Drug Abuse Grant P30 DA023026 (RHH) and the National Institute on Aging Grants P30 AG034424 (VJH) and P30 AG066619 (KAG). The funders had no role in study design, data collection and analysis, decision to publish, or preparation of the manuscript.

We hope you find the revised manuscript suitable for publication in *PLOS ONE*.

Thank you.

Sincerely yours,

Rick H. Hoyle, Ph.D.

Professor

**EDITOR SUGGESTIONS**

Thank you for the careful review of our manuscript and the invitation to submit a revised version. We are delighted to hear that you believe our work has merit and hope that our edits and revisions will fully meet *PLOS ONE*’s publication criteria.

***Journal requirements:***

When submitting your revision, we need you to address these additional requirements.

1. Please ensure that your manuscript meets PLOS ONE's style requirements, including those for file naming.
   a. Thank you for the note about these requirements. The files have been updated to appropriately match the journal requirements.
2. Thank you for stating the following financial disclosure:

“This work was funded by the National Institute on Drug Abuse Grant P30 DA023026 (RHH) and the National Institute on Aging Grants P30 AG034424 (VJH) and P30 AG066619 (KAG).”

Please state what role the funders took in the study.

If the funders had no role, please state: "The funders had no role in study design, data collection and analysis, decision to publish, or preparation of the manuscript." If this statement is not correct you must amend it as needed.

Please include this amended Role of Funder statement in your cover letter; we will change the online submission form on your behalf.

a. We have included an amended Role of Funder statement in our cover letter.

3. We notice that your supplementary tables are included in the manuscript file. Please remove them and upload them with the file type 'Supporting Information'. Please ensure that each Supporting Information file has a legend listed in the manuscript after the references list.
   a. We have removed the supplementary tables and uploaded them with the appropriate file type and legend as requested.
4. Please review your reference list to ensure that it is complete and correct. If you have cited papers that have been retracted, please include the rationale for doing so in the manuscript text, or remove these references and replace them with relevant current references. Any changes to the reference list should be mentioned in the rebuttal letter that accompanies your revised manuscript. If you need to cite a retracted article, indicate the article’s retracted status in the References list and also include a citation and full reference for the retraction notice.
   a. We have made a thorough review of our references to ensure the list is complete and correct.

**REVIEWER SUGGESTIONS**

**Reviewer #1**:

Many thanks for the opportunity to review this interesting submission which I really enjoyed reading. Your submission demonstrates an excellent standard of writing and was clear and easy to read and follow. You might want to further explain the phrase used on p 11 line 249 "the right tails in the metrics" which may be unfamiliar to some readers. I have no further suggestions to improve.

Thank you for your kind words regarding our submission. We are delighted to hear that you enjoyed reading our work and that you felt our submission demonstrated an excellent standard of writing. We sincerely appreciate your note regarding the phrase “the right tail in the metrics” and have revised that statement as noted below. We believe this suggestion strengthened the clarity of this portion of our results.

“The differences in median values of time gaps, distance gaps, and the number of participation days were much smaller across urban and non-urban locations than differences in mean values, which were influenced by a small number of high values on these metrics.”

**Reviewer #2**:

This is a useful report on how to record location + survey data from urban and rural settings remotely, to identify and analyze activity spaces. This approach can be useful for research in various fields. Congratulations for the clarity in the manuscript, and for providing open access to fieldwork protocols and data. Some minor comments:

Thank you for your careful review of our work and your kind note about the clarity of the manuscript. We sincerely appreciate your comments and suggestions, which helped us produce a revised version that is clearer and more informative. Responses to each suggestion are included below.

1. Authors listed ‘intensive longitudinal design’ as a keyword; however, other than the fact that the data came from a larger longitudinal study, no reference to the “intensive longitudinal design” was made in the manuscript. I’d consider removing this from the list.
   a. We have removed this phrase from the list of keywords.
2. It could be useful to see a brief background of studies that have focused in urban studies and some if they exist who have analyzed areas beyond cities, and especially in rural settings. Especially, authors could provide a brief description of limitations/challenges identified in those studies, if any.
   a. Thank you for this suggestion. We agree that further exploration of prior research in urban studies is warranted in our introduction. We have revised the original text and added some additional text as noted below.

(pg. 1) Revisions (in purple) & additions (in red)

“People may travel out of and potentially far from their residential space for errands, obligations, work, or social engagements. Thus, activity spaces include residential areas but also extend beyond them. The distance people travel, as well as the spaces they visit, vary in terms of geographic, structural, cultural, and social features. Intuitively, urban and non-urban spaces are likely to differ because of these features; however, a recent review of urban mobility research noted that simultaneous comparisons of these activity spaces are currently lacking [1].”

(pg. 2) Revisions (in purple) & additions (in red)

“In prior urban studies of activity space, some additional challenges have been identified, such as the possibility that tracking may lead participants to alter their behavior, the potential for one week to be insufficient for identifying behavioral patterns, and the broader issue of being unable to capture the heterogenous experiences of people in different neighborhood and social contexts [1]. Despite these challenges, each study adds to the emerging activity space literature and is crucial for addressing these limitations. We implemented this method in non-urban contexts to address the lack of activity space research within non-urban areas [1] and identify potential implementation challenges.”

3. In table 1, why is the number of participation days so low, with values around 0.5? Also, why is temporal coverage median 7 – maybe these two variables are reversed in the table?
   a. We appreciate the careful review of our tables. These variables labels had been reversed as you suggested. We have updated the table and replaced “Temporal coverage” with “Number of days participated” and replaced “Number of days participated” with “Temporal coverage” (pg. 12).

Thank you.

Thank you again for your comments!
